# Abacavir-Reactive Memory T Cells Are Present in Drug Naïve Individuals

**DOI:** 10.1371/journal.pone.0117160

**Published:** 2015-02-12

**Authors:** Andrew Lucas, Michaela Lucas, Anette Strhyn, Niamh M. Keane, Elizabeth McKinnon, Rebecca Pavlos, Ellen M. Moran, Viola Meyer-Pannwitt, Silvana Gaudieri, Lloyd D’Orsogna, Spyros Kalams, David A. Ostrov, Søren Buus, Bjoern Peters, Simon Mallal, Elizabeth Phillips

**Affiliations:** 1 Institute for Immunology and Infectious Diseases, Murdoch University, Perth, Australia; 2 Laboratory of Experimental Immunology, Faculty of Health Sciences, University of Copenhagen, Copenhagen, Denmark; 3 School of Anatomy, Physiology & Human Biology, University of Western Australia, Nedlands, Australia; 4 Department of Clinical Immunology & Immunogenetics, Royal Perth Hospital & Pathwest, Perth, Australia; 5 School of Pathology & Laboratory Medicine, University of Western Australia, Nedlands, Australia; 6 Vanderbilt University School of Medicine, Nashville, Tennessee, United States of America; 7 Department of Pathology, Immunology and Laboratory Medicine, University of Florida College of Medicine, Gainesville, Florida, United States of America; 8 Division of Vaccine Discovery, La Jolla Institute for Allergy and Immunology, La Jolla, California, United States of America; University of Montreal Hospital Research Center (CRCHUM), CANADA

## Abstract

**Background:**

Fifty-five percent of individuals with HLA-B*57:01 exposed to the antiretroviral drug abacavir develop a hypersensitivity reaction (HSR) that has been attributed to naïve T-cell responses to neo-antigen generated by the drug. Immunologically confirmed abacavir HSR can manifest clinically in less than 48 hours following first exposure suggesting that, at least in some cases, abacavir HSR is due to re-stimulation of a pre-existing memory T-cell population rather than priming of a high frequency naïve T-cell population.

**Methods:**

To determine whether a pre-existing abacavir reactive memory T-cell population contributes to early abacavir HSR symptoms, we studied the abacavir specific naïve or memory T-cell response using HLA-B*57:01 positive HSR patients or healthy controls using ELISpot assay, intra-cellular cytokine staining and tetramer labelling.

**Results:**

Abacavir reactive CD8+ T-cell responses were detected *in vitro* in one hundred percent of abacavir unexposed HLA-B*57:01 positive healthy donors. Abacavir-specific CD8+ T cells from such donors can be expanded from sorted memory, and sorted naïve, CD8+ T cells without need for autologous CD4+ T cells.

**Conclusions:**

We propose that these pre-existing abacavir-reactive memory CD8+ T-cell responses must have been primed by earlier exposure to another foreign antigen and that these T cells cross-react with an abacavir-HLA-B*57:01-endogenous peptide ligand complex, in keeping with the model of heterologous immunity proposed in transplant rejection.

## Introduction

Abacavir hypersensitivity reaction (HSR) is a potentially life threatening CD8+ T cell mediated, HLA-B*57:01 restricted syndrome previously occurring in 5–8% of those treated with the drug, but now prevented by HLA-B*57:01 screening prior to abacavir prescription [1–11]. Abacavir HSR has occurred exclusively in those carrying the HLA-B*57:01 allele and patients carrying related B17 serotype alleles such as HLA-B*58:01 and HLA-B*57:03 are known to be tolerant of abacavir. Recently, the structural basis of the restriction of abacavir HSR to HLA-B*57:01 has been determined and reveals that abacavir binds non-covalently and specifically within the antigen-binding groove of HLA-B*57:01. Abacavir forms contacts within the deep hydrophobic F-pocket of the groove which effects the shape and chemistry of the antigen binding cleft and consequently alters the repertoire of HLA-B*57:01-restricted peptides presented to CD8+ T cells [12,13]. This abrupt change in the peptide repertoire is analogous to what occurs in organ transplantation where immune recognition of neo-antigen results in graft rejection. In this context, pre-existing Class I restricted effector memory CD8+ T cells which have specificities to prevalent or persistent viruses may cross recognize an HLA mismatched allograph [14]. The rapidity of such CD8+ T-cell responses is enhanced by the higher precursor frequency of the antigen specific cells and their lack of requirements for co-stimulation or CD4+ T-cell help. This contrasts with requirements necessary to prime and expand a naïve T-cell response [14,15]. Similarly, we propose that immunity to abacavir results from cross-reactive memory CD8+ T cells previously primed by past immune experience, and possibly also naïve CD8+ T cells primed by drug dependent neo-antigen(s).

Immunologically confirmed abacavir HSR only occurs in individuals with the HLA-B*57:01 allele and this 100% negative predictive value has been crucial to the success and implementation of HLA-B*57:01 as a routine screening tool to prevent abacavir HSR. However, only 55% of individuals with HLA-B*57:01 exposed to the drug will develop hypersensitivity [3]. We and others have shown that abacavir reactive CD8+ T cells can be consistently expanded following culture from 100% of HLA-B*57:01 positive unexposed donors but never from HLA-B*57:01 negative donors. The *in vitro* findings are therefore compatible with the 100% negative predictive value of the test but not the 55% positive predictive value. Furthermore, the onset of abacavir HSR symptoms can occur as early as 36 hours after first exposure, characteristic of re-activation of pre-existing memory T cells but also as late as 3 weeks, which is more characteristic of either a delayed expansion of pre-existing memory CD8+ T cells or with the *in vivo* expansion of naïve CD8+ T-cell responses. Here we report findings that support the contribution of both mechanisms; we detect abacavir responsive CD8+ T cells *ex vivo* within PBMC from HLA-B*57:01 positive abacavir-unexposed donors and also demonstrate that abacavir can drive the expansion of CD8+ T-cell responses from both sorted naïve or memory T cells from HLA-B*57:01 positive donors. We therefore propose a model in which an HLA-B*57:01 restricted CD8+ memory T-cell response to a currently unknown pathogen specific epitope cross-recognizes an endogenous peptide that is only presented by HLA-B*57:01 in the presence of pharmacological levels of abacavir. Exploiting the fact that vaccination and immunity to yellow fever is not prevalent in the developed world, we demonstrate that within the yellow fever vaccine response of a HLA B*57:01 positive individual we can detect a breadth of CD8+ T-cell clonotypes that recognise both the yellow fever wildtype KF9 epitope and synthetic variants of this epitope that can only be presented in an abacavir treatment dependent manner. We offer this is as “proof in principle” of an altered peptide repertoire / heterologous immunity model in which the abacavir associated shift in self-peptide presentation contains self-peptide(s) that can cross-react with a pre-existing memory T-cell population.

## Methods

The authors acknowledge the recently published Minimum Information about T cell Assays (MIATA) Guidelines [16,17] and have provided detailed information in accordance with MIATA.

### Classification of clinical defined HSR patients

Unpublished data from the PREDICT-1 study [3] was analyzed to determine days to abacavir HSR symptoms onset in patch test confirmed cases.

### Primary cells and Cell lines

Healthy or HIV+ donors were selected on the basis of their carriage or not of the HLA-B*57:01 allele. PBMC were separated by density centrifugation (Histopaque-1077 or ficoll; Sigma-Aldrich) and cryopreserved by suspension at 1 × 10^7^/ml in 90% FCS/10% DMSO, transferred into-1C/min freezing containers (Mr Frosty, Thermo Fisher) at-80 C and then transferred to vapour-phase liquid nitrogen storage, at -180°C until use. Cell numbers and viability were evaluated using an automated cell counter (Countess, Life Technologies) and average cell yield following thawing was typically 9 × 10^6^ PBMC with 95% viability. The HLA-B*57:01-transfected, MHC-I-deficient B lymphoblastoid cell line C1R.B57 [2] was cultured with geneticin (Sigma-Aldrich) and washed before use.

IFN-γ ELISpot assay: Interferon-γ ELISpot assays were performed as previously described [18], with the following modifications. ELISpot plates (MAIPS4510; Millipore) were coated with 15 μg/ml anti-IFN-γ antibody (Mabtech) and used to assay 2 × 10^5^ viable cells in triplicate. Assay plates were evaluated using an automated plate reader (AID Plate Reader Version 5.0, AID GmbH).

### Culture and detection of abacavir responsive T cells

Abacavir responsive short term lines were produced from cryopreserved PBMC, as previously described [2]. To enumerate abacavir responsive T cells, C1R.B57 antigen presenting cells (APC) were cultured overnight ± 10 μg/mL abacavir (pure substance; GSK), then washed. Treated or untreated C1R.B57 APC were added at a 1:10 ratio to PBMC and incubated at 37°C /5% CO_2_ for six hours, in the presence of 20 μg/mL Brefeldin-A (Sigma-Aldrich) for the final four hours. Samples were surface labelled for CD4 and CD8 expression on ice and then fixed and permeabilized for the detection of intra-cellular IFN-γ, as previously described [2]. Abacavir responsive cells were then detected using flow cytometry (Gallios; Beckman Coulter), as follows. Lymphocytes were gated on the basis of forward and side scatter properties. The lymphocyte gate was then gated for CD8 positivity but not CD4 positivity using a Boolean logic gate and the frequency of CD8 and IFN-γ double positive cells was determined using Kaluza software (Beckman Coulter).

### Interferon-γ capture assay

Interferon-γ (IFN-γ) positive abacavir reactive cells were detected in PBMC from abacavir unexposed donors following stimulation with C1R.B57 APC treated ± abacavir, as described above, for four hours. Cells were then labelled with an IFN-γ capture reagent and IFN-γ was captured for forty-five minutes, according to manufacturer’s directions (Miltenyi Biotec). Cells were then washed and labelled with anti-IFN-γ-PE, anti-CD3-Pacific Blue and anti-CD8-APC-H7 (Becton Dickinson). Cells were either analyzed immediately by flow cytometry or following enrichment of positive cells using anti-PE microbeads and magnetic column separation (Miltenyi Biotec) and then analyzed by flow cytometry [19]. A lymphocyte gate was set using FSC and SSC parameters. CD3+ lymphocytes were then analysed for CD8 and IFN-γ expression. Positive gates were set above the level of the background staining of anti- IFN-γ-PE on unstimulated CD3+ PBMC.

### Cell sorting of Naïve and Memory T cell subsets

Cryopreserved PBMC samples were labelled using CD4-PE, CD8-APC-Cy7, CD45RA-APC (all Becton Dickinson) and CD62L-ECD (Beckman Coulter) on ice, following overnight rest to restore CD62L expression (Figure A in S1 File). CD4+ and CD8+ gated lymphocytes were sorted (Influx; Becton Dickinson) on the basis of CD45RA and CD62L expression [20]. Sort purities were regularly >98%. We confirmed naïve and memory populations by stimulating naïve or memory CD8+ T cells with cytomegalovirus (CMV), Epstein-Barr virus (EBV) and influenza derived peptides (CEF peptides; Mabtech) and performing an IFN-γ ELISpot assay as described above. CEF peptides responses were only detected in stimulated memory phenotype T cells, but not naïve populations (Figure B in S1 File).

### Production and staining with abacavir independent and dependent B*57:01 tetramers

The yellow fever specific peptide epitope K9F (KTWGKNLVF) and two synthetic variants, K9A (KTWGKNLVA) and K9V (KTWGKNLVV), were synthesized by standard 9-fluorenylmethyloxycarbonyl (FMOC) chemistry and purified by reversed-phase high-performance liquid chromatography (purity at least 80%, usually >95%) (Schafer-N, Copenhagen, Denmark). Tetramers were produced as previously described [21] with the following modification: abacavir (ABC; 200 μg/ml) was added to the folding reaction for the production of the variant tetramers, K9A-ABC-B*57:01 and K9V-ABC-B*57:01. Successfully folding of MHC-I monomers with these peptides was strictly abacavir dependent. To tetramerize the complexes Streptavidin-R-Phycoerythin (PE), Streptavidin-Allophycocyanin (APC), or Streptavidin-Brilliant Violet 421 (BV421) (Biolegend, San Diego, USA) was sequentially added over 60 min at a molar ratio of Streptavidin to peptide-MHC-I monomer of 1:4.

### Cell staining

PBMC’s from an HLA-B*57:01 positive donor drawn day 17 post yellow fever vaccinated (17D-204; Sanofi-Pasteur) were expanded with the K9F epitope. Cells were harvested day 7 and stained with K9F-B*57:01-PE, K9A-ABC-B*57:01-APC, and K9V-ABC-B*57:01-BV421 tetramers at RT for 20min, and subsequently washed and stained for CD3 and CD8 for 30 min. at 4°C. The cells were subsequently analyzed by flow cytometry (Fortessa; BD Biosciences).

*Statistics*: Positivity of abacavir ELISpot responses was defined using a cut-off response of ≥ 10 SFU/10^6^ PBMC above background, and differences in proportions of positive responses were assessed using a Fisher’s exact test. A donor-stratified Wilcoxon test was used to assess paired differences in magnitude of responses across restimulated abacavir specific cell line cultures, p values of less <0.05 were considered significant. Statistical testing was not carried out if the sample size was less than five.

### General Laboratory Operations

The Institute for Immunology and Infectious Diseases (IIID) laboratories at Murdoch University are accredited by the Australian National Association of Testing Authorities (NATA). All assays are performed according to standard operating procedures that are managed using a document control system (Q-Pulse). Laboratory staff training is regularly updated and documented by Q-Pulse. Experimental samples and data are tracked through the laboratory using a Laboratory Information Management Systems (LIMS) and data and sample integrity is protected.

### Ethics Statement

Murdoch University, Royal Perth Hospital and Australian Bone Marrow Donor Registry ethics committees approved the study that was conducted according to the principles expressed in the Declaration of Helsinki. All study participants provided informed written consent prior to their participation. A patient consent form approved by the Murdoch University, Royal Perth Hospital and Australian Bone Marrow Donor Registry ethics committees was used to document consent.

## Results

### Immunologically confirmed abacavir HSR occurs less than two days following first exposure to abacavir

**Fig 1:**
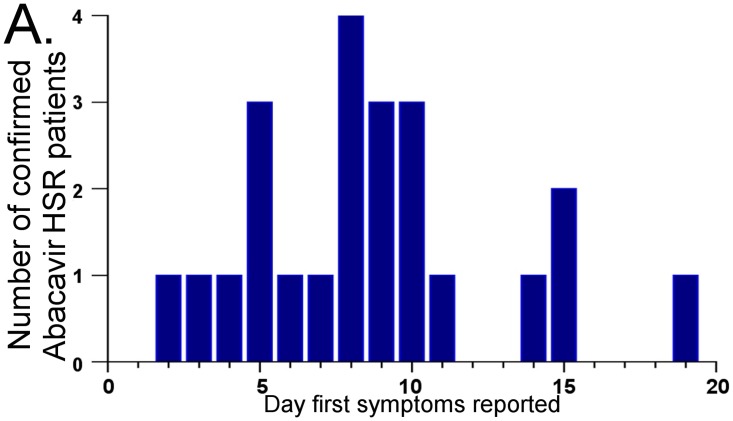
Days to onset of hypersensitivity symptoms in patch test confirmed cases of abacavir HSR (range <2–19 days, median 8 days; n = 23).

Abacavir patch testing is a specific and highly sensitive research tool used to identify HLA-B*57:01 positive patients who have experienced immunologically-mediated abacavir HSR [3,11,22]. This test was utilized in the randomized, controlled and double-blinded PREDICT-1 study where none of the 1956 individuals enrolled had symptoms of hypersensitivity and became patch test positive in the arm that underwent real-time HLA-B*57:01 screening. In the unscreened arm of the study there were 23 subjects with a clinical diagnosis of abacavir HSR who became patch test positive and all 23 carried HLA-B*57:01. Here we show further that these patch test positive patients all had onset of symptoms within 19 days, with 26% having onset within 36 hours to five days following first drug exposure (Fig. 1, S1 Table). Abacavir HSR symptom onset as early as 36 hours following first exposure suggests that cross-reactive memory CD8+ T-cell responses are responsible at least in some individuals. The study had a double-blind design and an independent expert panel adjudicated the patch test photographs to eliminate the possibility of bias.

### Abacavir hypersensitive patients demonstrate abacavir-reactive memory T-cell responses, detectable years following primary drug exposure

**Fig 2:**
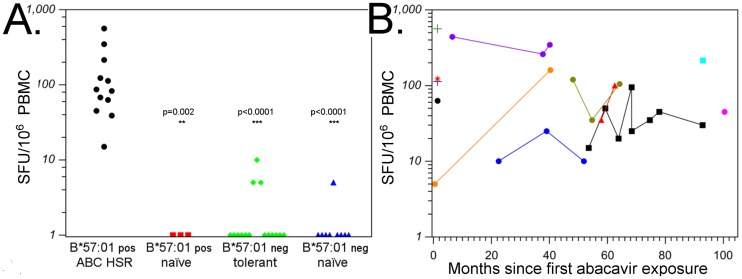
Abacavir ELISpot responses. **A**. Mean abacavir ELISpot responses for HLA B*57:01 positive or negative individuals, that were either abacavir naïve or treatment experienced. **B** Abacavir ELISpot responses in patch test confirmed abacavir HSR patients plotted relative to time from first exposure to abacavir (25-562 SFU/10^6^ PBMC, n = 12). Positivity of abacavir ELISpot responses was defined using a cut-off response of ≥ 10 SFU/10^6^ PBMC above background, and differences in proportions of positive responses were assessed using a Fishers exact test.

Non-covalent binding of abacavir to HLA-B*57:01 results in the presentation of self-peptides that would not otherwise be presented [12,13] suggesting that drug associated neo-antigen exposure should have rapid onset and offset. Supporting this, symptoms of acute abacavir HSR correlate with plasma level and disappear within 24–48 hours of drug withdrawal. To assess whether this transient exposure to abacavir leads to establishment of long-term memory, we measured overnight IFN-γ ELISpot assay responses of 12 patients with patch test confirmed abacavir HSR (Part A in S2 Table). We compared these responses to those from HLA-B*57:01 positive abacavir naive patients, HLA-B*57:01 negative tolerant or abacavir naïve patients (Parts B & C in S2 Table). The HLA-B*57:01 positive abacavir HSR patients show significantly higher responses when compared to each control group (Fig. 2A). In particular 12/12 elicited positive responses in contrast to the controls (0/3 HLA-B*57:01 positive drug naïve controls, p = 0.002; 1/15 HLA-B*57:01 negative abacavir tolerant control, p<0.0001; 0/9 HLA-B*57:01 negative drug naïve controls, p<0.0001; Fisher exact test). Moreover, the magnitude of ELISpot responses measured at multiple time-points in the same individual indicates the maintenance of a durable CD8+ T-cell response (Fig. 2B).

### A low frequency population of CD8+ T cells from HLA-B*57:01 abacavir-unexposed subjects respond to abacavir ex vivo

**Fig 3:**
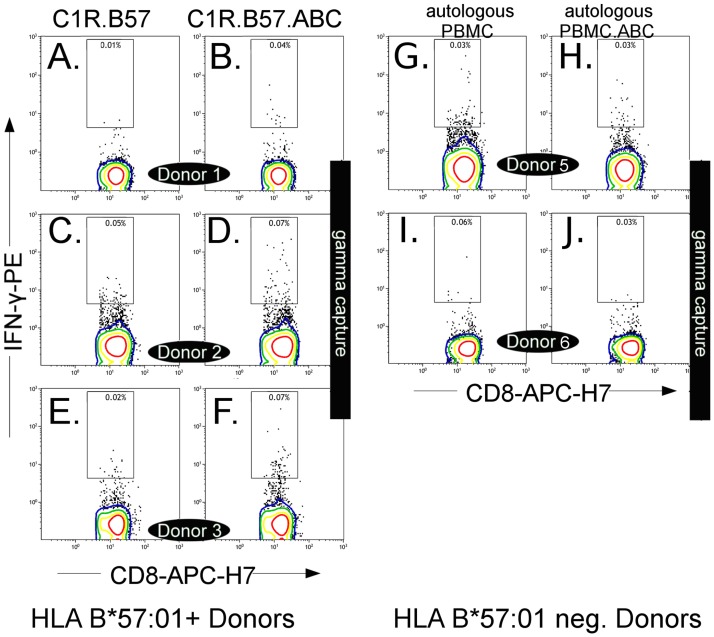
Abacavir responsive CD8+ T cells detected in HLA-B*57:01+ abacavir-unexposed donors. HLA-B*57:01+ (n = 3) from abacavir unexposed donors were stimulated for 5h with 1:10 C1R.B57 APC (left column) or abacavir treated C1R.B57 APC (right column), and CD8+/ IFN-γ+ cells detected by IFN-γ capture assay and enumerated by flow cytometry. Cells that fall within the depicted gates are considered positive. Donors 1 (A, B) and 2 (C, D) are the results from cryopreserved PBMC. Donor 3 (E, F) is the result of a freshly separated PBMC sample. Similarly, HLA-B*57:01 negative PBMC (n = 2) from abacavir unexposed donors were stimulated for 5h with 1:10 fractions of autologous PBMC (PBMC: left column) or autologous PBMC treated with abacavir (PBMC.ABC: right column) and CD8+/IFN-γ cell detected by IFN-γ capture assay and enumerated by flow cytometry. Cells that fall within the depicted gates are considered positive. Donor 4 (G, H) and Donor 5 (I, J) are the results from cryopreserved PBMC.

We examined whether the rapid and early clinical responses to abacavir in some patients could be explained by expansion of pre-existing memory CD8+ T cells by exposing PBMC from abacavir-unexposed HLA-B*57:01 positive (n = 3) and negative donors (n = 2) to APC stimulation with either the HLA-B*57:01 transfected single HLA antigen cell line C1R.B57 or autologous PBMC, ± abacavir (Fig. 3). We used a five hour IFN-γ capture assay to measure IFN-γ responses. A low frequency of abacavir responsive CD8+/IFN-γ+ T cells were detected within PBMC from three of three abacavir unexposed HLA-B*57:01 positive donors stimulated with abacavir treated C1R.B57 APC (Fig. 3: right column) compared to untreated C1R.B57 (Fig. 3: left column). The abacavir responsive CD8+/IFN-γ+ T cells were detectable above background in two cryopreserved HLA-B*57:01 donor samples (Fig. 3: A compared to B, C compared to D) and also in freshly separated PBMC (Fig. 3: E compared to F). No comparable increase in CD8+ IFN-γ + T cells were detected above background in two of two HLA-B*57:01 negative donors stimulated with abacavir treated autologous PBMC (Fig. 3: G compared to H and I compared to J).

### Abacavir responsive CD8+ T cells can be expanded from HLA-B*57:01 positive PBMC and from sorted memory or naïve T cells from the same donors

**Fig 4:**
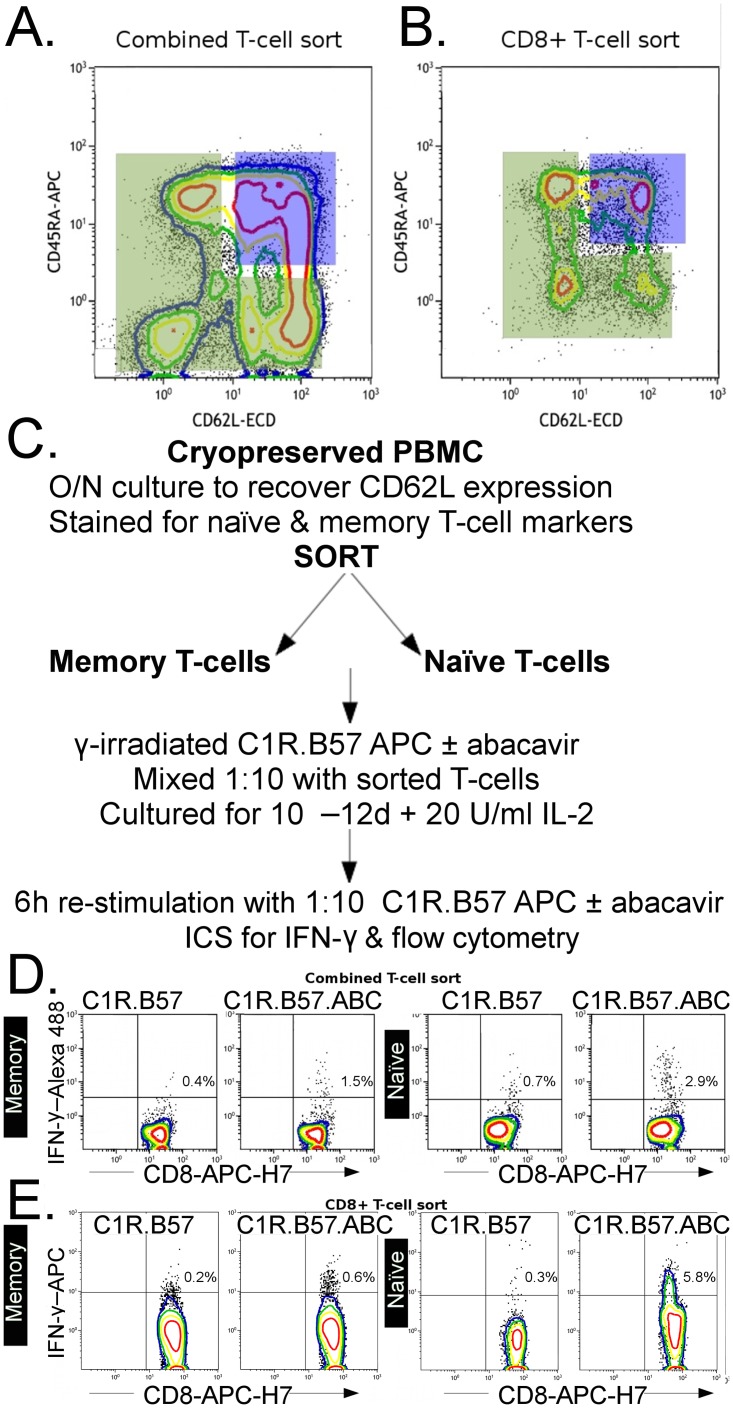
Assessment of abacavir responsive memory or naïve T cells. **A**. Sorting gates used to collect total CD4+ and CD8+ memory phenotype (green polygon) and naïve phenotype T cells (blue rectangle) or **B**. CD8+ memory or naïve phenotype T cells only. **C**. Protocol used for the selection, priming and re-stimulation of sorted naïve and memory T cells from cryopreserved HLA-B*57:01 positive donor PBMC. **D**. Representative plots of in-vitro cultures of HLA-B*57:01 positive memory (left most pairs of plots) or naïve (right most pairs of plots) phenotype T cells derived from sorting strategy A. Cultures were re-stimulated with APCs treated with abacavir (C1R.B57.ABC) or untreated (C1R.B57), respectively. **E**. Representative plots of in-vitro cultures of HLA-B*57:01 positive memory (left most pairs of plots) or naïve (right most pairs of plots) phenotype CD8+ T cells derived from sort strategy B. Cultures were re-stimulated with APCs as described above.

We have previously demonstrated that robust abacavir treatment specific CD8+ T-cell responses can be reproducibly induced in *in vitro* 10 day cultures of PBMC from drug naïve HLA B*57:01 positive donors (2). We have used this culture protocol to examine the contributions of pre-existing memory T cells, and/or naïve T cells in the establishment of abacavir associated immunity, using abacavir-unexposed HLA-B*57:01 positive and negative PBMC from healthy donors, which included three of the four donors examined in Fig. 3. We examined the abacavir specific response of unsorted donor PBMC or sorted memory (Fig. 4A: Sort gate shaded green), or sorted naïve (Fig. 4A: Sort gate shaded blue) T cells or sorted CD8+ memory (Fig. 4B: Sort gate shaded green) or sorted CD8+ naïve T cells Fig. 4B: Sort gate shaded blue). Cultured cells were stimulated with irradiated C1R.B57 APC treated with abacavir (C1R.B57.ABC) or untreated (C1R.B57) and cultured for 10 days. At that time aliquots of the cultures were re-stimulated with similarly treated APC to detect IFN-γ production from abacavir responsive CD8+ T cells as shown in Fig. 4C. Representative responses for memory and naïve cultures derived from a CD4+ and CD8+ T-cell sort (Fig. 4D) or a CD8+ T-cell sort (Fig. 4E) are shown.

**Fig 5:**
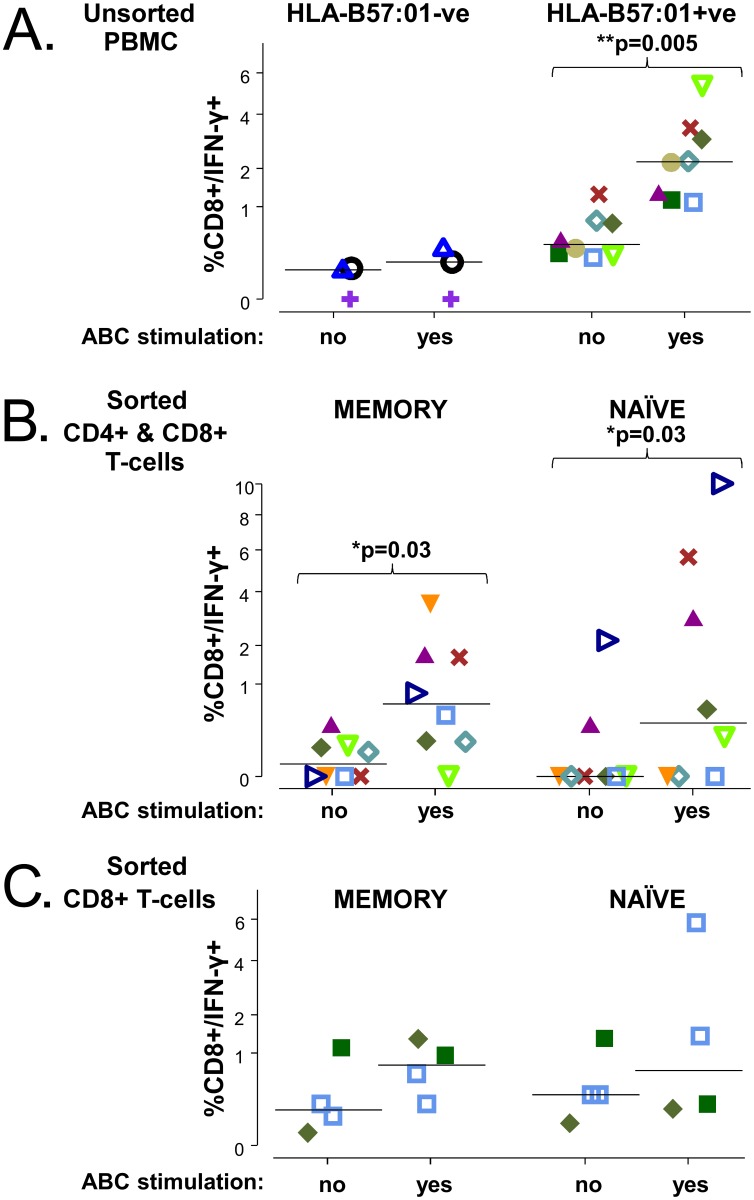
Abacavir responsive CD8+ T cells can be expanded from unsorted, memory and naïve phenotype T cells from abacavir-unexposed HLA-B*57:01 positive donors. CD8+/IFN-γ frequencies are compared across T-cell populations which had been cultured ± abacavir, and then re-stimulated with APCs treated with abacavir (yes) or untreated (no), respectively. **A**: CD8+/IFN-γ frequencies in unsorted PBMC from HLA-B*57:01 negative (n = 3) and positive donors (n = 8). CD8+/IFN-γ frequencies in HLA-B*57:01 positive donors according to memory and naïve phenotype in **B**: sorted CD4+ and CD8+ T cells (n = 8) and **C**: sorted CD8+ T cells only (4 samples, n = 3 donors). Each donor is represented by the same symbol in the different figures; group median CD8+ /IFN-γ+ T-cell frequencies are indicated by a horizontal line. Pairwise differences in observed frequencies according to abacavir stimulation are assessed by a donor-stratified Wilcoxon test.

Short-term abacavir responsive T-cell lines were generated from all eight unsorted PBMC samples tested. Similar to previous reports [2,23], we could detect abacavir reactive CD8+/IFN-γ+ T-cells in 100% of eight HLA-B*57:01 positive donors (2.21% [1.26–3.12] versus 0.35% [0.23–0.69]; median [IQR] p = 0.005; Fig. 5A) and in 0% of three HLA-B*57:01 negative donors (0.16% [0.08–0.23] versus 0.10 [0.05–0.11]; median [IQR], not significant.

These results also confirmed the HLA-B*57:01 restriction of the CD8+ T-cell responses induced in the presence of abacavir [1,2]. The *in vitro* expansion of abacavir specific CD8+ T-cell responses in 100% (8/8) of HLA-B*57:01 positive healthy donors is higher than the 55% incidence of HSR seen in HLA-B*57:01 positive patients exposed to abacavir in the PREDICT-1 trial [3] (p = 0.01, exact Binomial test).

To formally examine the contribution of naïve or pre-existing memory T cells to the abacavir reactive CD8+ T cells detected above, we sorted pure populations of both from a selection of these donors, primed them with APC (C1R.B57 APC for HLA-B*57:01 positive cells and autologous PBMC for HLA-B*57:01 negative donors) with or without abacavir pre-treatment, cultured them for 10–12 days in the presence of IL-2 and then re-stimulated the cultures with the same APC combinations. In cultures derived from sorted memory T cells there were significantly higher %CD8+/ IFN-γ T cells detected when re-stimulated with C1R.B57.ABC APC compared to untreated C1R.B57 APC (0.63% [0.15–1.67] versus 0.04% [0.00–0.10]; median [IQR], p = 0.03, Fig. 5B. In some, but not all, cultures derived from sorted naïve T cells there were much higher %CD8+/ IFN-γ +T cells detected when re-stimulated with C1R.B57.ABC APC compared to untreated C1R.B57 APC (0.36% [0.00–3.58] versus 0.00% [0.00–0.08] median [IQR], p = 0.03 (Fig. 5B).

### Abacavir responsive CD8+ T cells can be expanded from sorted HLA-B*57:01 positive memory and naïve CD8+ T cells, in the absence of CD4+ T cells

To determine whether there was a requirement for CD4+ T cells in the expansion of abacavir responsive CD8+ T cells, we sorted memory or naïve CD8+ T cells (n = 4, from three donors), which were then stimulated, cultured for 10 days, and then re-stimulated, as described in Fig. 4C. In cultures derived from sorted memory CD8+ T cells, we detected slightly higher frequencies of CD8+ IFN-γ+ T cells when they were re-stimulated with C1R.B57.ABC APC compared to untreated C1R.B57 APC (0.77% [0.50–1.03] versus 0.15% [0.08–0.43], median [IQR], non-significant). Similarly, in cultures derived from sorted naïve CD8+ T cells we also detected higher frequencies of abacavir responsive CD8+ IFN-γ+ T cells when cultures were re-stimulated with abacavir treated APC (C1R.B57.ABC) than untreated APC (C1R.B57) (0.80% [0.18–1.50] versus 0.30% [0.24–0.56], median [IQR], non-significant). Taken together, we conclude that, at least in some individuals, there is no requirement for the presence of CD4+ T cells to expand abacavir specific cells in culture.

### Vaccination can induce CD8+ T-cell populations that cross-recognize an HLA-B*57:01 restricted viral vaccine epitope and abacavir-dependent variant epitopes

To support the idea that the CD8+ T cells, which can be expanded in the presence of abacavir, could, at least in part, be derived from pre-existing memory T cells, we developed a model system involving human CD8+ T cell responses against a viral target, a live-attenuated yellow fever virus vaccine. A panel of yellow fever virus vaccinees, were used to identify a dominant HLA-B*57:01-restricted, yellow fever-specific CD8+ T cell epitope, K9F (KTWGKNLVF). With frequencies ranging from 0.2–0.6% of the CD8+ T cells, and 1–8% of the activated CD38+, CD8+ T cells, all yellow fever vaccinated HLA-B*57:01 positive donors (n = 6) recognized this wild type K9F epitope [24]. We then examined two of the donors to ascertain whether these wild type responses, induced by a viral challenge in the absence of abacavir, included CD8+ T cell responses that could cross-react with K9F variants, which could only be presented in the presence of abacavir. To this end, synthetic variants of the K9F epitope were generated containing valine or alanine instead of a phenylalanine at P9 of the epitope. These variant peptides only bound to HLA-B*57:01 in the presence of abacavir [12]. Thus, it was only possible to produce HLA B*57:01 tetramers with these variant peptides if the peptide-HLA-B*57:01 complexes were generated in the presence of abacavir. These “variant, abacavir-dependent” tetramers, as well as wild-type K9F, HLA-B*57:01 tetramers generated in the absence of abacavir, were prepared with different fluorochromes allowing us to use the different tetramers in combinations to probe the abacavir-dependent cross-reactivities of the wild type yellow fever specific CD8+ T-cell response.

**Fig 6:**
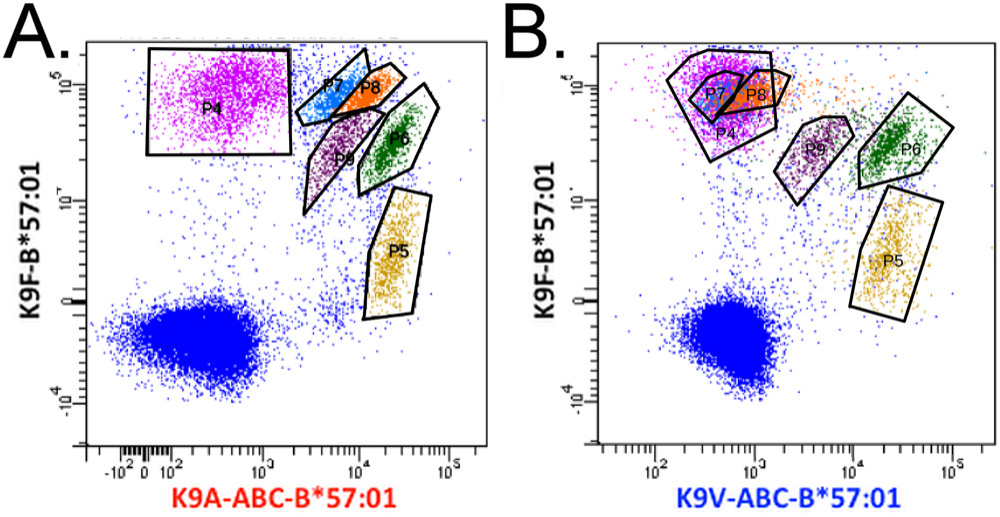
Immunization of an HLA-B*57:01 donor with the yellow fever vaccine results in the expansion of populations of CD8+ T cells that detect both the wild type epitope and abacavir dependent variant epitopes. T cells expanded from a yellow fever vaccinated B*57:01 donor were labelled with anti-CD3 and CD8 and a mixture of K9F-B*57:01-PE, K9A-ABC-B*57:01-APC, and K9V-ABC-B*57:01-BV421 tetramers. CD8+ T cells were gated and the combination of K9F-B*57:01-PE and K9A-ABC-B*57:01-APC tetramer stained CD8+ T cells are shown in A. Subpopulations of tetramer stained cells are gated: P4 (pink); P5 (yellow); P6 (green); P7 (blue); P8 (orange); P9 (purple). The combinations of K9F-B*57:01-PE and K9V-ABC-B*57:01-BV421 tetramer stained CD8+ T cells are shown in B. The colors identify the position of the gated tetramer stained populations in panel A.

In both donors, one of which is shown in Fig. 6, the K9F-B*57:01 CD8+ T-cell response was polyclonal and could be separated into at least six populations based on T-cell cross-recognition of the abacavir bound variants, K9A-ABC-B*57:01 and K9V-ABC-B*57:01. The first population (gate P4; Fig. 6) recognized only the K9F epitope and none of the variants. The second and third populations (gate P7 and P8; Fig. 6) recognized the K9F epitope with a high staining intensity and cross-recognized the K9A-ABC-B*57:01 variant with an intermediate staining intensity, but not the K9V-ABC-B*57:01 variant. The fourth population (gate P9; Fig. 6) recognized the K9F epitope with an intermediate staining intensity and cross-recognized both the K9A-ABC-B*57:01 and the K9V-ABC-B*57:01 variant with intermediate staining intensities. The fifth population (gate P6; Fig. 6) recognized the K9F epitope with an intermediate staining intensity and cross-recognized both the K9A-ABC-B*57:01 and the K9V-ABC-B*57:01 variant with high staining intensities. The sixth population (gate P5; Fig. 6) recognized the K9F epitope with a low staining intensity and cross-recognized both the K9A-ABC-B*57:01 and the K9V-ABC-B*57:01 variant with intermediate staining intensities.

## Discussion

Abacavir HSR and its association with HLA-B*57:01 is the strongest and most specific of any described immunologically-mediated adverse drug reaction to-date. The absolute restriction of abacavir HSR susceptibility to HLA-B*57:01 positive patients is related to the specific non-covalent binding of abacavir along the floor and into the F-pocket of the antigen binding groove, which dramatically alters the peptide binding characteristics of HLA-B*57:01 resulting in a different set of peptides being selected for loading onto the HLA class I molecule.

Our findings support a role for pre-existing memory responses in the development of abacavir HSR. Development of symptoms of abacavir HSR within 36 hours to five days of first drug exposure is consistent with activation of a pre-existing memory response by an abacavir dependent neo-antigen. Here we describe that five hours following abacavir exposure we detect small numbers of pre-existing abacavir reactive CD8+ T cells within PBMC samples obtained from drug unexposed HLA-B*57:01 positive donors but not HLA-B*57:01 negative donors, a result we attribute to memory T cells present in these donors (Fig. 3). We acknowledge the low number of donors that we were able to test in this experiment as a limitation. However, consistent and in support of this observation, we were able to demonstrate a statistically significant expansion of abacavir responsive CD8+ T cells from sorted memory T cells from similar abacavir unexposed HLA-B*57:01 positive donors (Fig. 5). Additionally we were able to demonstrate a more variable but still statistically significant expansion of abacavir responsive CD8+ T cells from sorted naïve T cells from the same donors. The magnitude of the expansion of abacavir responsive CD8+ T cells from the sorted populations was reduced relative to the levels of expansion detected in all HLA-B*57:01 positive PBMC, cultured similarly from the same donors. Although there has been a recent report describing abacavir specific T cells in drug naïve susceptible healthy donors [23], these abacavir reactive CD8+ T cells were detected only following expansion in culture of PBMC for 14 days in the presence of abacavir, consistent with earlier work [2], and similarly confirmed here.

Abacavir binding to HLA-B*57:01 favors the presentation of peptides with an aliphatic C-terminal residue such as valine, alanine or isoleucine rather than the aromatic residues tryptophan or phenylalanine preferred by HLA-B*57:01. We describe a novel HLA- B*57:01 restricted epitope derived from Yellow Fever, K9F and exploited the knowledge of the effect of abacavir on peptide selection to produce C-terminal variants of the epitope containing either an alanine (K9A) or valine (K9V). We generated MHC-I tetramers containing the wild type K9F peptide and MHC-I tetramers containing the K9A or K9V peptides that could be folded only in the presence of abacavir. In both HLA-B*57:01 positive, yellow fever vaccinated donors who were examined, we were able to show that the donor’s memory responses included a breadth of CD8+ T cell clonotypes that included T-cell receptors (TCR) which exclusively recognized the K9F epitopes but importantly also TCR which recognized both the K9F epitope and which also cross-recognized the synthetic variants of the epitope with an alanine (K9A) or valine (K9V) at the P9 position of the epitope.

The intent of these experiments was a proof of concept to demonstrate that abacavir can support cross-reacting T-cell responses. We were able to prove this by identifying multiple sub-populations of CD8+ T cells that could recognize an HLA-B*57:01 restricted viral epitope and cross-recognize synthetic epitopes that could only be presented by HLA-B*57:01 in the presence of abacavir.

Thus, the CD8+ memory T cells induced after yellow fever vaccination include subsets of T-cells with specificities that cross-recognize several different structurally related, variant peptides, which can only be presented by HLA-B*57:01 in the presence of abacavir. By inference, any self-peptide, which, due to its lack of HLA-B*57:01 binding, has not been involved in establishing self-tolerance, but becomes presented in the presence of abacavir, could be cross-recognized by pre-existing CD8+ memory T cells directed against structurally related foreign peptides (e.g. of viral origin). This mechanism of abacavir-dependent cross-reactions is reminiscent of the “molecular mimicry” that has been invoked in the generation of autoimmune responses. We have previously noted that the alteration of the HLA-B*57:01 bound peptide repertoire caused by abacavir and the resulting massive activation of T cells has parallels with an alloreactive response (12). In combination, these two mechanisms may explain how abacavir can induce a large and diverse population of autoreactive T cells.

Similarly, we propose that pre-existing abacavir reactive CD8+ memory T cells are pathogen specific and HLA-B*57:01 restricted. Some of these memory T cells have the potential to cross-react with the many host cells which express abacavir dependent self-peptide/s bound to HLA B*57:01, but only when pharmacologic levels of abacavir are present. It follows that whilst the patient is receiving abacavir treatment, the abacavir dependent self-peptide epitope has the potential to be expressed on a much larger proportion of host cells than those infected by the priming pathogen, an effect which is rapidly lost following drug withdrawal as it is rapidly cleared.

Despite the 55% PPV of HLA-B*57:01 for abacavir HSR, we were able to expand abacavir reactive CD8+ T-cell responses *ex-vivo* from the PBMC of 100% of HLA-B*57:01 positive abacavir unexposed individuals. Since it is likely that HLA-B*57:01 positive abacavir tolerant patients will present the same wide range of novel endogenous epitopes as HSR patients, this suggests that only a subset of the abacavir reactive CD8+ T cells that are detected or expanded *in vitro* are mediating HSR in vivo [1,2,4,5,12,13]. This is analogous to the model of rejection of HLA- mismatched organ transplantation. In this model, broad allogeneic responses can be generated *in vitro* to a range of neo-antigens to which the T cells have not been tolerized. Only a subset of these allo-responses are believed to mediate organ rejection, and these responses are mediated by pre-existing effector memory CD8+ T cells specific to prevalent and/or persistent viruses [14]. Memory T-cell subsets have lower activation requirements and lower requirements for co-stimulation than naïve responses [25–27]. In the organ transplant rejection model, the mechanistic basis for cross-reactivity between an HLA-B8 restricted CD8+ T-cell response to the EBV epitope FLR and HLA-B*44:02 has been characterized and the propensity for TCR plasticity to result in heterologous immune response was described [28–30]. In keeping with a similar propensity for T-cell cross-reactivity in drug hypersensitivity, Adam et al have recently demonstrated alloreactivity in 5% of abacavir reactive clones from abacavir unexposed HLA-B*57:01 positive, healthy donors [31].

Whilst we were able to expand abacavir reactive CD8+ T cells from the sorted memory T cells of all our healthy HLA-B*57:01 positive abacavir unexposed individuals, this was true for only about 50% of sorted naïve T cells from the same individuals. The inefficiency of this process may be related to the limitations of our *in vitro* culture system. Alternatively, it has recently been reported that stem cell memory CD8+ T cell (T_SCM_), restricted to common recall antigens, express CD45A and CD62L+ [32] and we cannot exclude the possibility that some T_SCM_ may have been included along with truly naïve T cells in our sorts. Like conventional memory T cells, T_SCM_ respond to re-stimulation with cognate antigen rapidly, but have greater proliferative responses [32]. Inter-patient variability in the presence of such T_SCM_ which cross-react to an abacavir dependent self-antigen might explain the incomplete positive predictive value of clinical abacavir HSR, however we did not look for abacavir responsive CD62L+, CD45RA+, CD95+, CD8+ T cells in this study.

Other explanations can be offered to explain the incomplete positive predictive value of HLA-B*57:01 for abacavir HSR. Individuals may lack naïve T-cell specificities capable of responding to the new drug peptide target as has been recently demonstrated for HLA-B*15:02 associated Stevens-Johnson syndrome/toxic epidermal necrolysis where a specific TCR clonotype is prevalent amongst HLA-B*15:02 positive cases but not in HLA-B*15:02 positive carbamazepine tolerant controls [33]. Alternatively, but less likely, the peptide(s) which only binds the HLA allele in the presence of the drug may be expressed in the target cells of some people but not others due to either genetic polymorphisms in the coding regions of normal self-peptides or due to differences in protein expression levels in specific target tissues between individuals.

The non-covalent binding of drug to the MHC molecule resulting in presentation of an altered peptide repertoire provides a mechanistic basis for drug-MHC-peptide interactions and explains how drug hypersensitivity could be exclusively T-cell mediated. The additional contribution of heterologous immunity to this or other models of drug hypersensitivity would explain both the distribution and type of clinical manifestations and the sometimes very rapid onset of symptoms after drug initiation as well as whether or not a drug hypersensitivity syndrome will occur in a genetically predisposed individual. For example, following abacavir exposure in an HLA-B*57:01 positive individual, if cross-reactive CD8+ memory T cells exist in a high enough precursor frequency they will expand inducing rapid onset of symptoms within five days of first drug exposure. In other individuals it is possible that the contribution of these pre-existing cross-reactive memory CD8+ T-cell responses may be slow to expand or weaker than the priming of naïve CD8+ T-cell responses against a broader range of abacavir dependent endogenous peptides, leading to the onset of HSR symptoms in the second to third week of abacavir treatment.

If this model is correct then it will be necessary to identify both the relevant specific HLA-restricted anti-pathogen response and the drug-specific self-peptide that the CD8+ T cells are cross-reacting to. T-cell responses to viruses, particularly the chronic persistent human herpes virus (HHV) infections, have been shown to mediate allogeneic organ rejection via TCR cross-reactivity (14, 28–30), and we propose a similar heterologous immunity model to explain T-cell mediated drug hypersensitivity. Our observation that T cells recognizing a yellow fever vaccine epitope cross-react with synthetic variants of the epitope that can only be presented by HLA-B*57:01 in the presence of abacavir provide a proof of principle for such a model. However, we emphasize that our experiments were designed only as a proof of principle to show that cross-reactivity can occur and yellow fever vaccination is not a pre-condition for abacavir hypersensitivity. *In vivo*, as in the transplantation model, HLA-B*57:01 restricted CD8+ T-cell responses to a highly prevalent and ubiquitous chronic persistent infection, such as one of the HHV, may prove to be the source of the cross-reactivity to the abacavir-induced neo-antigen. Characterizing the role of such heterologous immune responses in drug hypersensitivity may provide approaches to examine the role of heterologous immunity in other forms of autoimmune or allergic hypersensitivity.

## Supporting Information

S1 Table Clinical characteristics of patch test positive, HLA-B*57:01 positive subjects with abacavir hypersensitivity. (DOCX)

S2 Table Clinical characteristics of HIV positive subjects. (DOCX)

S1 File Supporting Figures. **Figure A**. CD62L expression on thawed cryopreserved peripheral blood mononuclear cells. **Figure B**. Sorted memory phenotype T cells respond to CEF peptide stimulation but naïve cells do not.
(DOCX)
