# Supplementary material for: Abacavir-Reactive Memory T Cells Are Present in Drug Naïve Individuals
Source: PLoS One. 2015 Feb 12;10(2):e0117160. doi: 10.1371/journal.pone.0117160 (PMC4326126; doi:10.1371/journal.pone.0117160)
Supplement: S1 Table — (DOCX) [file pone.0117160.s001.docx]

**Table S1**

| **Clinical Characteristics of Patch Test Positive, HLA-B*57:01 positive Abacavir Hypersensitivity Subjects** | | | | | | | |
| --- | --- | --- | --- | --- | --- | --- | --- |
| **ID** | **Age at sample** | **HLA B type (2^nd^ allele)** | **Race** | **CD4%** | **HIV load**  **(log)** | **CNNRTI*** | **Time to HSR** |
| 1 | 37 | 15:03 | white | 21 | n/a | N | 8 |
| 2 | 35 | 56:01 | white | 27 | 2.601 | N | 9 |
| 3 | 36 | 15:01 | white | 33 | 2.601 | N | 5 |
| 9 | 44 | 44:02 | white | 29 | 2.601 | N | 9 |
| 5 | 41 | 51:01 | white | 18 | 2.601 | N | 8 |
| 6 | 38 | 14:02 | white | 16 | 4.322 | N | 5 |
| 7 | 58 | 08:01 | white | 15 | 5.2401 | N | 8 |
| 8 | 35 | 44:03 | white | 20 | 3.9445 | N | 11 |
| 9 | 40 | 44:03 | white | 18 | 5.1139 | N | 8 |
| 10 | 48 | 44:03 | white | 12 | 5.3802 | Y | 10 |
| 11 | 38 | 07:02 | white | 34 | 2.601 | N | 5 |
| 12 | 45 | 40:02 | white | 15 | 2.601 | N | 4 |
| 13 | 40 | 35:01 | white | 33 | 2.601 | N | 7 |
| 14 | 44 | 35:03 | white | 14 | 2.601 | N | 2 |
| 15 | 24 | 52:01 | white | 49 | 2.601 | Y | 9 |
| 16 | 32 | 40:01 | white | 3 | 4.9912 | N | 6 |
| 17 | 63 | 27:05 | white | 24 | 3.9138 | N | 10 |
| 18 | 31 | 18:02 | white | 8 | 5.6021 | N | 19 |
| 19 | 42 | 38:01 | white | 35 | 3.6532 | Y | 15 |
| 20 | 48 | 15:01 | white | 44 | 2.601 | N | 10 |
| 21 | 31 | 08:01 | black | 22 | 2.601 | N | 14 |
| 22 | 42 | 41:01 | white | 23 | 2.601 | N | 3 |
| 23 | 48 | 51:08 | white | 21 | 2.601 | N | 15 |

*CNNRTI = concurrent non-nucleoside reverse transcriptase inhibitor
