# Supplementary material for: Abacavir-Reactive Memory T Cells Are Present in Drug Naïve Individuals
Source: PLoS One. 2015 Feb 12;10(2):e0117160. doi: 10.1371/journal.pone.0117160 (PMC4326126; doi:10.1371/journal.pone.0117160)
Supplement: S2 Table — (DOCX) [file pone.0117160.s002.docx]

**Table S2**

| **Clinical Characteristics of HIV Positive Subjects** | | | | | |
| --- | --- | --- | --- | --- | --- |
| - HLA-B*57:01 **positive** patch test positive abacavir HSR (Part A) | | | | | |
| **ID** | **Age at sample** | **HLA B type** | **Race** | **CD4 count (/μl)** | **HIV viral load**  **(copies/ml)** |
| HSR-1 | 56 | 51, 5701 | White | 231 | 72 |
| HSR-2 | 39 | 5701, 4001 | White | 425 | <50 |
| HSR-3 | 46 | 0702, 5701 | White | 270 | <50 |
| HSR-4 | 47 | 08, 5701 | White | 160 | 1000 |
| HSR-5 | 37 | 5701 | White | ND | <50 |
| HSR-6 | 50 | 0801, 5701 | White | 378 | <50 |
| HSR-7 | 69 | 4001, 5701 | White | 560 | <50 |
| HSR-8 | 59 | 5701, 5501 | White | 240 | <50 |
| HSR-9 | 46 | 57, 44 | White | 874 | <50 |
| HSR-10 | 54 | 5701, 44 | White | 924 | 87 |
| HSR-11 | 63 | 4001, 5701 | White | 270 | <50 |
| HSR-12 | 49 | 5701, 4403 | White | 616 | <50 |
| - HLA-B*57:01 **negative** abacavir tolerant (Part B) | | | | | |
| **ID** | **Age at sample** | **HLA B type** | **Race** | **CD4 count** | **HIV load** |
| Tol-1 | 32 | 3530, 3802 | SE Asian | 56 | 56 |
| Tol-2 | 42 | 58, 18 | SE Asian | 456 | 456 |
| Tol-3 | 49 | 3501, 3906 | White | 1080 | 1080 |
| Tol-4 | 45 | 3501, 5001 | White | 357 | 357 |
| Tol-5 | 47 | 27, 44 | White | 714 | 714 |
| Tol-6 | 54 | 0702, 1302 | White | 1056 | 1056 |
| Tol-7 | 42 | 4002, 4403 | White | 630 | 630 |
| Tol-8 | 42 | 0702, 4101 | White | 660 | 660 |
| Tol-9 | 60 | 5101, 0801 | White | 323 | 323 |
| Tol-10 | 43 | 08, 16 | White | 644 | 644 |
| Tol-11 | 40 | 4001, 4403 | White | 810 | 810 |
| Tol-12 | 34 | 3906, 44 | White | 14 | 14 |
| Tol-13 | 64 | 3501, 5701 | White | 528 | 528 |
| Tol-14 | 45 | 3501, 4002 | White | 48 | 48 |
| Tol-15 | 51 | 5501, 3501 | White | 342 | 342 |
| - HLA-B*57:01 **positive** abacavir naïve (Part C) | | | | | |
| **ID** | **Age at sample** | **HLA B type** | **Race** | **CD4 count** | **HIV load** |
| B57pos-Naïve-1 | 44 | 1401, 5701 | White | 725 | <50 |
| B57pos-Naïve-2 | 55 | 5701, 3701 | White | 891 | <50 |
| B57pos-Naïve-3 | 49 | 5701,4403 | White | ND | ND |

*in addition n=9 HLA-B*57:01, HIV negative, abacavir unexposed healthy donors were used as controls as shown in Part A

+Part B include ELISpots from multiple time points from subjects HSR-1 through HSR-12
