# Supplementary material for: Abacavir-Reactive Memory T Cells Are Present in Drug Naïve Individuals
Source: PLoS One. 2015 Feb 12;10(2):e0117160. doi: 10.1371/journal.pone.0117160 (PMC4326126; doi:10.1371/journal.pone.0117160)
Supplement: S1 File — Figure A. CD62L expression on thawed cryopreserved peripheral blood mononuclear cells. Figure B. Sorted memory phenotype T cells respond to CEF peptide stimulation but naïve cells do not. (DOCX) [file pone.0117160.s003.docx]

**S1 File**
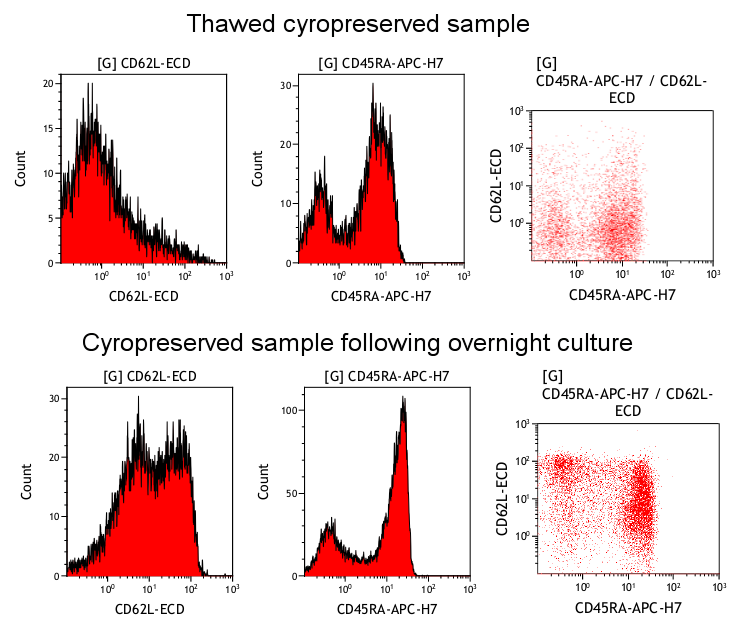
**Figure A**

CD62L expression recovered by overnight culture.

It has been commonly reported that lymphocyte expression of CD62L is lost when PBMC are cryopreserved. We examined whether the expression of this marker might recover after placing a thawed PBMC in culture. The top 3 panels show the histogram staining for CD62L and CD45RA contrasting the low to negative CD62L expression and the clearly CD45RA positive population. The dot plot of both markers clearly shows a failure to detect the double positive CD62L+/CD45RA+ naive T-cell populations under these staining conditions.

In contrast, following overnight culture of an aliquot of the same sample in RPMI-1640 media containing 10% FCS and antibiotics clear bimodal CD62L staining, similar CD45RA expression and CD62L+/CD45RA+ naive T-cells were all detected.


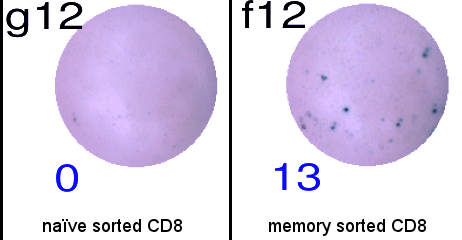


**Figure B** Sorted Memory phenotype T cells respond to CEF peptide stimulation but naïve T cells do not.

Sorted Memory and Naive phenotype T cells were plated at cell numbers representative of their frequency in 200000 unsorted PBMC. Responses to CEF peptides were tested by overnight IFN-γ ELISpot assay. Memory but not Naive sorted T cells showed clear responses to the CEF peptide stimulation.
